# Use of Percutaneous Cholecystostomy for the Management of Complicated Cholecystitis Causing Gastric Outlet Obstruction in an Elderly Patient

**DOI:** 10.7759/cureus.39708

**Published:** 2023-05-30

**Authors:** Hannah Jones, Dylan Murray, Richard Murray, Mohamed Elfedaly

**Affiliations:** 1 Department of Surgery, Texas Tech University Health Sciences Center, Amarillo, USA; 2 Department of Surgery, University College Dublin, Dublin, IRL; 3 Department of Radiology, Texas Tech University Health Sciences Center, Amarillo, USA

**Keywords:** subtotal cholecystectomy, interval cholecystectomy, percutaneous cholecystostomy tube, complicated cholecystitis, chronic cholecystitis, gastric outlet obstruction (goo)

## Abstract

The management of complicated cholecystitis in an elderly patient can present a complex clinical decision for surgeons. There is literature supporting the use of immediate laparoscopic cholecystectomy for cases of uncomplicated cholecystitis in elderly patients and complicated cholecystitis in the general population. There are, however, no clear guidelines for treating the unique presentation of an elderly patient with complicated cholecystitis. This is likely due to the many clinical risk factors that must be considered when caring for these complex patients often with many medical comorbidities. In this report, we present the case of an 81-year-old male with complicated chronic cholecystitis leading to the exceedingly rare complication of gastric outlet obstruction. The patient was successfully treated with percutaneous cholecystostomy tube placement and interval subtotal laparoscopic cholecystectomy.

## Introduction

Acute cholecystitis is a common condition that sends nearly 700,000 patients in the United States to the operating room each year [1]. Laparoscopic cholecystectomy (LC) is considered the standard of care for cases of acute cholecystitis, and studies have shown that early intervention with laparoscopic cholecystectomy decreases the risk of readmission, postoperative sepsis, and the need for conversion to an open procedure [2,3]. While LC has a well-established low risk of complications, elderly or acutely ill patients with various comorbidities increase the risks associated with emergency surgery [4]. For these patients at high surgical risk, percutaneous cholecystostomy tube placement (PC) with interval LC has been regarded as a safe alternative to emergency surgery [5]. The literature presents conflicting evidence for the care of these high-risk patients with gallbladder disease, and the decision remains up to the clinical judgment of the surgeon [6-7]. In this report, we present a case of an elderly patient with severe complicated cholecystitis leading to a rare sequela of gastric outlet obstruction, successfully treated with PC and subsequent interval subtotal laparoscopic cholecystectomy.

## Case presentation

An 81-year-old man presented to the emergency department with hematemesis, weakness, nausea, and right upper quadrant and epigastric pain beginning 24 hours prior. He reported a one-year history of intermittent episodes of emesis after meals which began shortly after having a "stomach bug". During this time, he reported a 40-pound weight loss which he attributed to low caloric intake due to frequent emesis. His past medical history was significant for gastroesophageal reflux, hiatal hernia, nephrolithiasis, thoracic and ascending aortic aneurysms, hypertension, and prostate cancer in remission. Surgical history was significant for right hip arthroplasty. Home medications included pantoprazole, celecoxib, oxybutynin, losartan potassium, tamsulosin, and atorvastatin. On arrival, vital signs were stable with a temperature of 98.6℉, heart rate of 80 bpm, respiratory rate of 18/min, and a moderately elevated blood pressure of 167/90. Laboratory results were significant for leukocytosis (15.5 K/uL) along with elevated total bilirubin (1.19 mg/dL), alkaline phosphatase (292 U/L), and lactic acid (2.2 mmol/L). The patient was admitted for suspected acute cholecystitis and started on intravenous broad-spectrum antibiotics.

**Figure 1:**
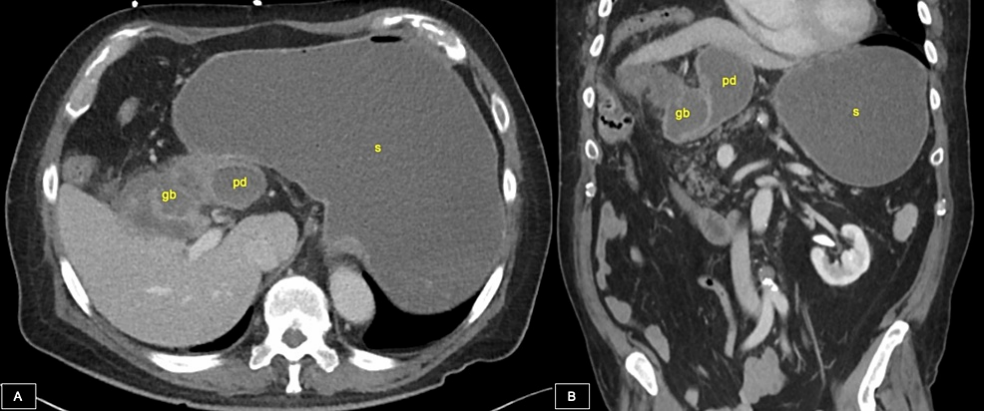
Computed tomography (CT) imaging findings 1a: Axial CT imaging of the abdomen with intravenous (IV) contrast showing massively distended stomach (s) and proximal duodenum (pd) with irregular gallbladder (gb) closely approximated to the proximal duodenum. 1b: Coronal CT imaging of the abdomen with IV contrast demonstrating compression of the proximal duodenum (pd) by the gallbladder.

Computed tomography (CT) (Figure 1) imaging revealed a massively distended esophagus, stomach, and proximal duodenum. The gallbladder appeared irregular suggestive of a possible history of chronic cholecystitis, and there was adhesion to and compression of the proximal duodenum. A nasogastric (NG) tube was placed, instantly draining approximately 2 liters of black fluid. The patient reported relief of epigastric pain immediately following decompression. Gastroenterology was consulted, and an endoscopy performed on day two of admission revealed mild compression of the duodenum with narrowing of the bulbar and post-bulbar regions and no presence of masses or ulcers.

To aid in management, magnetic resonance cholangiopancreatography (MRCP) was performed on day three of admission, which revealed a complex, multiloculated cystic collection in the right upper quadrant measuring approximately 8 cm. The gallbladder appeared to be approximately 5.1 cm, with thickened walls and central fluid intensity compressing the proximal duodenum. The diagnosis now favored severe, complicated cholecystitis with pericholecystic abscess leading to secondary gastric outlet obstruction.

On day four of admission, management options were weighed between performing a diagnostic laparoscopy or proceeding with conservative management with interventional radiology (IR)-guided percutaneous cholecystostomy drain placement. After careful review of imaging and evaluation of the complexity of the case, including late presentation, significant inflammation at the gallbladder fossa, and the presence of gastric outlet obstruction, it was determined to proceed with IR-guided cholecystostomy drain placement with a plan for interval cholecystectomy after the patient's condition improved.

Ultrasound-guided drainage yielded 110cc of frank pus, and gallbladder aspirate revealed acute inflammation with debris, hemorrhage, and bile pigment and no evidence of malignant cells.

By day seven of admission, the patient reported his pain had resolved, and he was tolerating an advancing diet with regular bowel movements. He was discharged home with a plan for removal of the drain and cholecystectomy in six weeks. The patient was seen in the outpatient clinic two weeks after discharge, and CT imaging demonstrated a resolving abscess.

Six weeks after the placement of the percutaneous cholecystostomy drain, the patient returned for robotic laparoscopic-assisted cholecystectomy. During surgery, the stomach, duodenum, entire transverse colon, and greater omentum were found to be adhered to the gallbladder. Due to the massive amount of adhesions and necrosis of the gallbladder, a critical view of vessels could not be obtained. A subtotal fenestrating cholecystectomy was deemed to be the safest intervention, and the gallbladder was amputated above the cystic plate using Bovie electrocautery. Endoscopic retrograde cholangiopancreatography (ERCP) was performed intraoperatively due to visualization of bile leak from the cystic duct, and sphincterotomy was performed along with stent placement. The surgery was prolonged, taking nearly four hours to complete; however, recovery was routine, and the patient was discharged home two days later. Final pathology showed florid chronic with focal acute inflammatory infiltrate and supplemental ulcerated mucosal epithelium with no evidence of malignant cells.

The patient returned eight weeks after surgery for a follow-up ERCP with stent removal. ERCP demonstrated a dilated common bile duct with no evidence of bile leak. Biliary sludge was present along with two small stones, which were cleared with a balloon sweep. The patient was discharged home the same day.

## Discussion

Management of elderly patients presenting with the acute need for surgery in the setting of multiple comorbidities presents a complex clinical decision for surgeons. In the setting of acute cholecystitis, most studies have shown equivocal or improved outcomes for elderly patients treated with immediate LC rather than PC with interval LC [6]. One systematic review showed most patients had initial clinical improvement of symptoms with PC (90.7%); however, mortality rates in patients treated with PC rather than with LC at the time of presentation were higher (15.4% vs. 4.5%) [6]. A meta-analysis analyzing the use of PC in elderly patients also reported mortality rates to be significantly higher in those treated with PC rather than immediate LC (POR=4.85; 95% CI: 1.02-7.20; p=0.0001) [8]. It should be noted that these studies were reporting based on the use of PC alone rather than the use of PC with subsequent LC. Only around 40% of elderly patients treated with PC will go on to have definitive treatment with subsequent LC [6,8]. The meta-analysis did report when patients were treated with PC and subsequent LC, the mortality rate was quite low (0.96%) [8]. Additionally, some degree of bias must be considered when interpreting these statistics, as those treated with PC are likely more acutely ill than those treated with LC at the time of presentation. One study reports in patients with pericholecystic fluid collection, such as that seen in our patient, those who received PC rather than immediate LC for acute cholecystitis had more favorable outcomes [9].

The presence of acute gastric outlet obstruction increased the complexity of the management of our patient. Significant adhesions and inflammation to the extent of causing gastric outlet obstruction put the patient at risk for duodenal perforation or injury had we proceeded with diagnostic laparoscopy at the time of presentation. Gastric outlet obstruction is an exceptionally unusual sequela of cholecystitis most often cited to be due to gallstone ileus secondary to cholecystoenteric fistula formation, known as Bouveret syndrome [10]. Gastric outlet obstruction has very rarely been cited to be due to secondary compression of the duodenum, such as that seen in our patient. Our literature search identified only two case reports citing gastric outlet obstruction secondary to external compression of the duodenum due to cholecystic causes [11-12].

In this report, we present an interesting case of complicated cholecystitis with pericholecystic abscess and an exceedingly rare complication of secondary gastric outlet obstruction. PC was utilized to reduce compression on the duodenum and relieve gastric outlet obstruction. This decreased the patient's risk for duodenal injury during interval cholecystectomy. Severe adhesions in the abdominal cavity contributed to a much longer than standard operation time, and lack of visibility of vessels required a subtotal cholecystectomy to be performed to ensure the safety of the patient. Postoperatively the patient was at risk for biliary leak and the need for secondary intervention; however, our patient had an uneventful recovery and overall steady improvement throughout his course [13].

## Conclusions

This case highlights the importance of using clinical judgment when determining the best management for patients with unique presentations in the setting of multiple comorbidities. While this case adds to the body of evidence supporting the use of percutaneous cholecystostomy and interval cholecystectomy in elderly patients with complicated presentations, our conclusions are limited by the nature of reporting a single case. More research with larger cohorts is necessary to definitively establish the efficacy and safety of this approach for future patients. Our patient had multiple surgical risk factors, including advanced age, hypertension, and gastric outlet obstruction. The gastric outlet obstruction seen in this patient was caused by compression of the duodenum from a very enlarged and necrotic gallbladder and pericholecystic abscess, as evidenced by effacement of the duodenum seen on imaging. This placed the patient at significant risk for duodenal perforation had we chosen to proceed with surgery at the time of initial presentation. Our elderly patient's late presentation with significant medical comorbidities led us to choose a more conservative approach with percutaneous cholecystostomy and interval laparoscopic subtotal cholecystectomy resulting in successful treatment. 
